# Biological control and plant growth promotion properties of *Streptomyces albidoflavus* St-220 isolated from *Salvia miltiorrhiza* rhizosphere

**DOI:** 10.3389/fpls.2022.976813

**Published:** 2022-08-30

**Authors:** Yongxi Du, Tielin Wang, Jingyi Jiang, Yiheng Wang, Chaogeng Lv, Kai Sun, Jiahui Sun, Binbin Yan, Chuanzhi Kang, Lanping Guo, Luqi Huang

**Affiliations:** ^1^State Key Laboratory Breeding Base of Dao-di Herbs, National Resource Center for Chinese Materia Medica, China Academy of Chinese Medical Sciences, Beijng, China; ^2^College of Pharmacy, Nanjing University of Chinese Medicine, Nanjing, China; ^3^National Agricultural Technology Extension and Service Center, Beijing, China; ^4^Key Laboratory of Biology and Cultivation of Herb Medicine, Ministry of Agriculture and Rural Affairs, Beijing, China

**Keywords:** biocontrol agents, plant growth-promotion, *Streptomyces albidoflavus*, *Salvia miltiorrhiza*, root rot disease

## Abstract

Root rot disease caused by *Fusarium oxysporum* is a devastating disease of *Salvia miltiorrhiza* and dramatically affected the production and quality of *Sa. miltiorrhiza*. Besides the agricultural and chemical control, biocontrol agents can be utilized as an additional solution. In the present study, an actinomycete that highly inhibited *F. oxysporum* was isolated from rhizosphere soil and identified as based on morphological and molecular characteristics. Greenhouse assay proved that the strain had significant biological control effect against *Sa. miltiorrhiza* root rot disease and growth-promoting properties on *Sa. miltiorrhiza* seedlings. To elucidate the biocontrol and plant growth-promoting properties of St-220, we employed an analysis combining genome mining and metabolites detection. Our analyses based on genome sequence and bioassays revealed that the inhibitory activity of St-220 against *F. oxysporum* was associated with the production of enzymes targeting fungal cell wall and metabolites with antifungal activities. Strain St-220 possesses phosphate solubilization activity, nitrogen fixation activity, siderophore and indole-3-acetic acid production activity *in vitro*, which may promote the growth of *Sa. miltiorrhiza* seedlings. These results suggest that *St. albidoflavus* St-220 is a promising biocontrol agent and also a biofertilizer that could be used in the production of *Sa. miltiorrhiza*.

## Introduction

*Salvia miltiorrhiza* is a well-important traditional Chinese medicinal plant with terrific economic, social, and medicinal benefits (Su et al., 2015). Its dried root, called Danshen for its medicinal use, has been used for hundreds of years (Jiang et al., 2019), primarily for the treatment of various cardiovascular and cerebrovascular diseases in China and other Asia countries. In addition, *Sa. miltiorrhiza* is also used as a health-promotion food (Shi et al., 2019). To fit the large demand of Danshen, the planting areas of *Sa. miltiorrhiza* has reached to 100 thousand hectares in China by the year of 2020. However, the production of *Sa. miltiorrhiza* was severely limited by root rot disease caused by *Fusarium oxysporum.* The average incidence of *Sa. miltiorrhiza* root rot disease in China is 10% ~ 30%. Moreover, in some plots where the disease severely happened, the incidence could reach to 80%, causing irreversible losses to farmers (Wang et al., 2018a).

Currently, the root rot disease on *Sa. miltiorrhiza* cannot be effectively controlled by using physical and chemical methods (Ye et al., 2003). Additionally, the long-term overuse of fungicides has caused many adverse effects on environment, animal and human health, soil quality, and pathogen controlling (Wang et al., 2014, 2018a; Raza et al., 2017). Consequently, it is important and urgent to develop alternative methods and agents that are less toxic and more effective in controlling root rot. Utilization of functional microbes that not only antagonistic to phytopathogens but also friendly to environment is considered an economical and effective method to control root rot disease and improve plant health. The use of functional microorganisms and their biological products can provide growers an option to not only avoid the problem of chemical residues on plants and soil, but also to reduce pathogen resistance (Handelsman and Stabb, 1996; Abbas et al., 2020; Sun et al., 2020). Strains of *Streptomyces* are considered as biocontrol agents due to their production of various active compounds with agricultural applications. In addition, they are able to survive in harsh environments and colonize the root of plants belonging to multiple species including *Sa. miltiorrhiza* (Suárez-Moreno et al., 2019; Jose et al., 2021; Wu et al., 2021). Moreover, *Streptomyces* strains have multiple strategies to suppress fungal pathogens such like nutrients competition, cell wall degradation, virulence factors degradation and plant immunity induction (Chen et al., 2018). Certain *Streptomyces* can also improve nutrient absorption and in turn boost plant development by producing auxins, solubilizing inorganic phosphate, fixing nitrogen and other methods (Goudjal et al., 2013; Vijayabharathi et al., 2015; Liu et al., 2016; Raaijmakers and Mazzola, 2016; Jones and Elliot, 2017). *Streptomyces* SCA2-4 T, isolated from the rhizosphere soil of prickly pear (*Opuntia stricta*), exhibited a strong antagonistic activity against *F. oxysporum* f. sp. *cubense* tropical race 4 causing banana *Fusarium* wilt (Qi et al., 2021). *Streptomyces* NEAU-S7GS2 isolated from the root of soybean does not only prevent Sclerotinia stem rot of soybean, but also promotes the soybean growth (Liu et al., 2019). Therefore, *Streptomyces* species offers abundant resources of biofungicides or biofertilizers for agricultural usage (Liu et al., 2019).

In the present study, *St. albidoflavus* strain St-220 was isolated from the rhizosphere soil of *Sa. miltiorrhiza*, and was identified based on its morphological and molecular characteristics. Additionally, the plant growth-promoting activity and antifungal activity of St-220 was also evaluated *in vitro* and in greenhouse conditions. To demonstrate the antifungal and growth-promoting mechanisms, we carried out an analysis combining genome mining and metabolites detection based on the genome sequence of St-220.The pathways for synthesis of secondary metabolites including antibiotics and plant growth-promoting compounds were investigated, and genes encoding the antifungal enzymes were also predicted. These results provided essential and deep insights into the biocontrol properties of *St. albidoflavus* St-220.

## Materials and methods

### Actinomyces and *Fusarium* strains

*Salvia miltiorrhiza* along with the rhizosphere soil were collected from *Sa. miltiorrhiza* plantation in Laiwu City, Shandong Province, China (36°18′N 117°50′E). The rhizosphere soil of *Sa. miltiorrhiza* were obtained from the root surface. The isolation of actinomycetes was performed according to the methods described previously with modifications (Wang et al., 2021). Briefly, 10 ml of soil suspension containing 1 g rhizosphere soil and 10 ml sterile water was incubated in a shaker at 100 rpm for 30 min, then diluted into 10^−3^ g/ml, 10^−4^ g/ml, and 10^−5^ g/ml. Two hundred microliters of the diluted suspension were added to Gause’s agar medium (containing 2% soluble starch, 0.051% K_2_HPO_4_, 0.025% MgSO_4_, 0.001% FeSO_4_, and 2% Agar B, pH 7.2–7.4) amended with 20 μg mL^−1^ nalidixic acid, respectively, and cultured at 28°C. For purification, single colonies grown on the plates were separately transferred to another plates and then stored at −80°C in 20% glycerol. The phytopathogenic fungi *F. oxysporum* was isolated from plant tissues of *Sa. miltiorrhiza* with root rot disease collected from a field in Yuzhou, Henan, in August 2019.

### Antagonistic effects of *Streptomyces* strains on *Fusarium oxysporum*

The inhibition ability of *Streptomyces* against *F. oxysporum* was determined using the conventional improved scribe inoculation method (Chen et al., 2018). A mycelium plug of *F. oxysporum* in the center of potato dextrose agar (PDA) plates. *Streptomyces* strains were inoculated by streaking symmetrically at the two sides of the plug, 25 mm to the plate center. Petri dishes not inoculated with *Streptomyces* were used as controls, and three times each experiment was performed. After incubation for 5 ~ 7 days at 28°C, the colony diameters were measured, and the growth inhibition (GI) was calculated according to the following formula (Qi et al., 2019):


$$\mathrm{Growth}\;\mathrm{inhibition}\;\left(GI\right)=\left[\left(D-d\right)/D\right]\times 100\%$$

where D and d represented the diameters of fungal colonies on the control and treated plates, respectively.

### Control effect of St-220 on *Salvia miltiorrhiza* root rot disease in greenhouse condition

Before planting, *Sa. miltiorrhiza* seeds were soaked in 75% ethanol for 5 min, and then soaked in 5% bleach for 10 min for surface disinfection. After rinsed with sterile water for three times, the seeds were placed in a culture bottle with a sterile mixture of soil and vermiculite (2:1). To make inoculum, a mycelium plug of *F. oxysporum* was inoculated in PDA liquid culture and incubated in a dark shaker at 28°C 180 rpm for 10 days, then the culture was cloth-filtered and the flow-through was saved as spore suspension, which was then adjusted to 1 × 10^7^ cfu/ml for use. To make cell suspensions of strain St-220, 500 μl of glycerol suspension was inoculated in 500 ml Gause’s liquid medium and incubated at 28°C 160 rpm for 10 days. The two-leaf *Sa. miltiorrhiza* seedlings were inoculated by drenching with 10 ml inoculum of *F. oxysporum* (Fo), 10 ml St-220 cell suspension mixed with 10 ml inoculum of *F. oxysporum* (Fo + St), and 10 ml of sterile water (CK), respectively. The inoculated seedlings were grown in a growth chamber with temperature of 30°C/26°C, photoperiod of 12/12 h and 50% humidity. At 30 days after inoculation (DAI), disease symptoms were observed and evaluated using a severity scale: 0 for no symptoms; 1 was suffered disease symptoms less than 20% (only 1 leaf yellowing or wilting); 2 and 3 were plants suffering from disease symptoms in the range of 20%–40% (more than 2 but less than half of the leaves turn yellow or wither) and 40%–80%, respectively; 4 was *Sa. miltiorrhiza* showing severe disease symptoms with only the top 1 to 2 leaves being healthy; level 5 was plants that have died (Li et al., 2022). The disease index (DI) was calculated based on the formula $DI\left(\%\right)=\sum \left[\left(A\times B\right)\times 100\right]/\left(C\times 4\right)\times 100\text{,}$ where A is the disease scale (0, 1, 2, 3, 4, and 5), B is the number of seedlings at each level of the scale, and C is the total number of seedlings for each treatment. Disease incidence and control efficiency were calculated according to the following formulas:

$\mathrm{Disease incidence}\;\left(\%\right)=\frac{\left(\mathrm{number of yellow leaves}\right)}{\left(\mathrm{total plant leaves}\right)}\times 100$
$\begin{array}{cc} \begin{array}{l} \mathrm{Control}\;\\ \mathrm{efficiency}\left(\%\right) \end{array} & =\frac{\left(\begin{array}{l} DI\;\mathrm{of control group}-\\ DI\;\mathrm{of treatment group} \end{array}\right)}{\left(\;DI\;\mathrm{of control group}\right)}\times 100 \end{array}$ (Li et al., 2022).

Plant traits including fresh and dry weight of the root and shoot of the seedlings, and the diameter and length of the roots were measured at 30 DAI. Ten seedlings in five culture bottles were inoculated for each treatment, and the experiment was repeated for three times.

### *In vitro* assessment of plant growth promotion traits

To evaluate the growth-promoting properties, the Phosphate solubilization, biological nitrogen fixation, siderophore and indoleacetic acid (IAA) production of the St-220 strain was determined. For this purpose, St-220 was cultured in 100 ml Gause’s liquid medium for 5 days at 28°C in an orbital shaker (150 rpm.), and each assay was performed with three biological replicates for each strain.

#### Phosphate solubilization

An improved Pikovskaya (PVK) solid medium was used to evaluate the ability of strain St-220 on insoluble organic phosphate solubilization. The plate was inoculated with strain St-220 and kept at 28°C for 7 days. Positive phosphate solubilization was evident by a clear halo around strain St-220 (Gupta et al., 1994). Plates inoculated with sterile water were as control. The experiment was repeated three times.

#### Biological nitrogen fixation

Assay for nitrogen-fixing activity of the strains was performed according to a modified procedure described previously (Roy, 1958): strain St-220 colony was inoculated on nitrogen-free agar medium (Ashby’s Nitrogen-free medium) and then incubated at 28°C 7 days for 3 times. That the strain grew after three consecutive transfers indicated nitrogen fixation activity.

#### Siderophore production

Chrome azurol blue agar was used to assess siderophore production, and the pH was adjusted to 7.2 with KOH as suggested previously (Schwyn and Neilands, 1987). The presence of a yellow halo indicates the production of siderophores.

#### Indoleacetic acid production

The IAA production activity of St-220 was determined by the method of Salkowski colorimetry (Tang and Bonner, 1948). The activated St-220 was inoculated to 0.5 g/L Gause’s agar liquid medium containing tryptophan, and then cultured at 28°C, 150 r/min in a shaker for 7 days to obtain the fermentation broth. One milliliter of the broth was centrifuged at 12,000 rpm for 5 min, then the supernatant was mixed with 2 ml Salkowski reagent containing 15 ml concentrated H_2_SO_4_, 25 ml distilled water and 0.75 ml of 0.5 M FeCl_3_.6H_2_O (de Oliveira-Longatti et al., 2014).

After incubation in darkness at room temperature for 30 min, the mixture turned pink when IAA was generated. Serial dilutions of a standard IAA solution (0, 5, 10, 15, 20, 25, 30, 35, and 40 μg/ml) were used to construct the calibration plot (Abbasi et al., 2019).

### Plant growth-promotion experiments

Seeds of *Sa. miltiorrhiza* were disinfected as described previously, and then planted in pots (7 cm × 7 cm × 10 cm) containing 100 g of sterilized soil substrate (nutrient soil: vermiculite = 1:1) for germination. When grew two leaves, seedlings that are similar in height were selected for use. To make inoculum, strain St-220 was grown on Gause’s liquid medium and incubated at 28°C 160 rpm for 10 days, then the harvested cell suspension was adjusted to 1 × 10^7^ cfu/ml. For inoculation, 20 ml of St-220 inoculum were applied to each pot, and 20 ml of sterile water was separately applied as negative control. The seedlings were inoculated every 10 days until the plant traits were investigated. Five replicates for each treatment, and the experiment was repeated three times. All the pots were placed in growth chamber at 30/26°C and 12/12 h, 50% humidity. Plant traits including root and shoot fresh weight, total dry weight, length and diameter of the root was measured at 40 DAI.

### Sequencing, assembly, annotation, and bioinformatics analysis of the genome of St-220

#### DNA extraction

To obtain the genomic DNA, a single colony of St-220 was transferred to Gause’s liquid medium and then incubated at 28°C at 160 rpm for 5 days. The obtained cell suspension was then centrifuged and the supernatant was yield for DNA extraction by the SDS method (Lim et al., 2016). The DNA purity and quantity were examined by using Qubit^®^ 2.0 Fluorometer (Thermo Scientific). The 16 s rDNA of St-220 was sequenced and compared to existing databases for identification.

#### Sequencing, assembly, and annotation

The obtained genomic DNA of St-220 was used for the whole genome sequencing by using the Illumina NovaSeq PE150 sequencing platform at Novogene Technology Co., Ltd. (Beijing, China). A series of *de novo* assemblies were carried out with different software (SOAP, SPAdes; Li et al., 2008; Simpson et al., 2009; Bankevich et al., 2012). The protein coding genes (CDSs), rRNA and tRNA were predicted by Glimmer version 3.02, RNAmmer 1.2 and tRNA-scan-SE version 2.0, respectively, (Lowe and Eddy, 1997; Delcher et al., 2007; Lagesen et al., 2007). For gene annotation, BLAST searches was carried out in several databases including NCBI Non-redundant (NR), Clusters of Orthologous Groups (COG; Jensen et al., 2007), Pfam (Finn et al., 2014), Swiss-Prot (Zhou et al., 2021), Carbohydrate-Active enZYmes (CAZy; Zhang et al., 2018), Kyoto Encyclopedia of Genes and Genomes (KEGG; Kanehisa and Goto, 2006) and Gene Ontology (GO; Ashburner et al., 2000). The online software antiSMASH version 6.0 was employed to definite antibiotic and secondary metabolite gene clusters (Blin et al., 2021).

### Identification and characterizations of strain St-220

#### Phylogenetic analyses

For identification of St-220, a phylogenetic tree was constructed based on the 16S rDNA and five housekeeping genes (*atpD*, *gyrB*, *recA*, *rpoB*, and *trpB*) concatenated sequences. Multiple alignment of the sequences and construction of phylogenetic tree using maximum likelihood were generated by using Clustal X (Larkin et al., 2007) and PhyloSuitev1.2.2 (Zhang et al., 2020), respectively. Calculation of orthoANI values (orthologous average nucleotide identity) was performed by JSpeciesWS (Richter et al., 2016) and an online tool ANI-Blast (ANIb) Calculator. The ANIb values were used for assessing two strains are same species. A Genome-to-Genome Distance Calculator (GGDC) web server version 3.0 (Rigden and Fernández, 2022) was used to determine DNA–DNA hybridization (DDH) values *in silico.*

#### Cultural and morphological characterizations

The morphological characteristics of the St-220 strain were observed under scanning electron microscopy (SEM; model S-3400 N, Hitachi, Ltd., Tokyo, Japan) when grown on PDA medium for 14 days. The mycelium and substrate mycelium characteristics of St-220 were investigated after incubation at 28°C for 14 days on PDA and Gause’s agar medium, respectively.

### Statistical analysis

Statistical analyses including Student’s *t*-tests and ANOVA with Dunnett’s test were performed with R scripts. Difference was considered significant when the *p* value was <0.05.

## Results

### *In vitro* antagonistic effects of *streptomyces* strains against *Fusarium oxysporum*

**Figure 1:**
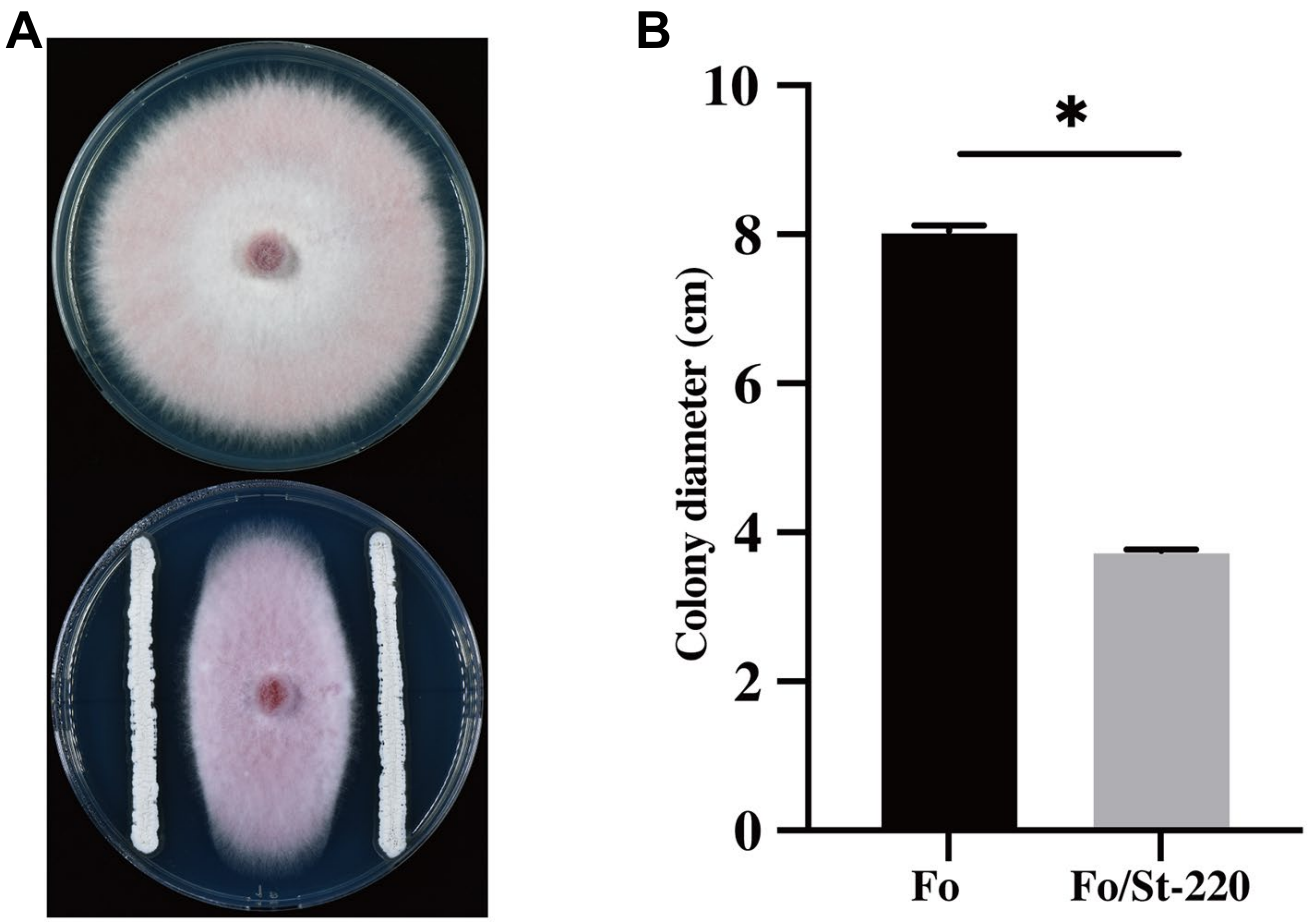
The antifungal activity of strain St-220 against *Fusarium oxysporum*. **(A)** *F. oxysporum* colony grew on PDA plate alone (upper) and with St-220 (lower) at 7 days after inoculation. **(B)** Colony diameter of *F. oxysporum* in each treatment. Bars with ∗ above are statistically different (*p* < 0.05).

A total of 163 strains of actinomycetes were isolated from the rhizosphere soil of *Sa. miltiorrhiza* in the plantation, and 11 strains showed an inhibitory effect on *F. oxysporum*, of which strain St-220 showed the most obvious inhibitory effect on mycelia growth of *F. oxysporum* (Supplementary Table S1). After 7 days incubation, the *F. oxysporum* incubated with St-220 showed narrow and oval colonies compared to the negative control (Figure 1A). To calculate the inhibition rate, the mean diameters of mycelia colonies were estimated by measuring the perpendicular length of each colony. The mean diameter of *F. oxysporum* mycelia colonies reached to 8.50 cm, while that of *F. oxysporum* grown with St-220 reached to 3.46 cm, with an inhibitory rate of 53.40% (Figure 1B).

### Control effect of St-220 on root rot disease of *Salvia miltiorrhiza* in greenhouse condition

**Figure 2:**
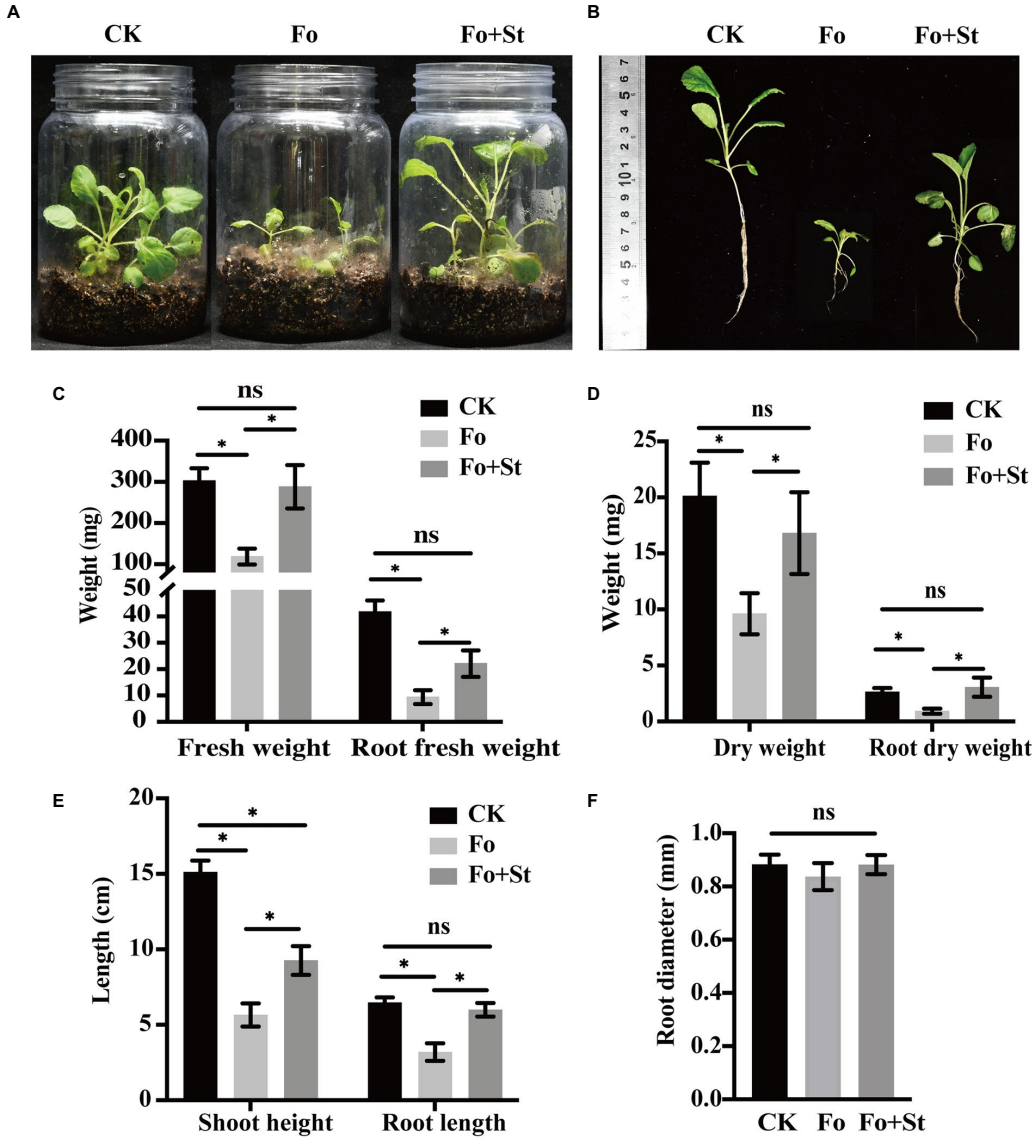
Control effect of St-220 on root rot disease of *Salvia miltiorrhiza* seedlings. **(A)** Symptoms of root rot developed on seedlings inoculated with *Fusarium oxysporum* (Fo) and mixture of *F. oxysporum* and St-220 (Fo + St) at 30 DAI, while no symptoms were observed on seedlings inoculated with sterile water (CK). **(B)** The entire plant of the seedlings in CK, Fo, and Fo + St treatment. Measurement of the fresh weight **(C)**, dry weight **(D)**, shoot height, root length **(E)**, and root diameter **(F)** of seedlings inoculated. Data are mean ± SE (*n* = 10). Means were compared with ANOVA analysis in combination with Tukey post-test. Means were considered statistically different when *p* < 0.05, Bars with ∗ above are statistically different, ns above are not statistically different.

After treated with cell suspension of strain St-220 for 30 days, *Sa. miltiorrhiza* seedlings in the pathogen treatment group displayed morphological indications of disease, with leaves turning yellow and wilting and roots rotting (Figure 2A). The disease incidence and disease index of the treatment group inoculated with *F. oxysporum* (Fo) were 86.67% and 68.00%, respectively, while the disease incidence and disease index of the treatment group inoculated with *F. oxysporum* and strain St-220 (Fo + St) were 20% and 22.66%, respectively. Strain St-220 significantly (*p* < 0.05) reduced disease incidence by 76.92% and disease index by 66.67% (Supplementary Table S2). Compared with the treatment Fo, the total fresh weight (Figure 2C), dry weight (Figure 2D), shoot height and root length (Figures 2B,E) of the Fo + St treatment significantly increased by 138.45%, 39.73%, 137.43%, and 72.12%, respectively. Meanwhile, root fresh weight, root dry weight, and root diameter were also increased (Figures 2C–F). Therefore, St-220 has the biological control impact on *Sa. miltiorrhiza* root rot in greenhouse condition.

### Biological characteristics involved in plant growth-promoting activity of St-220

**Figure 3:**
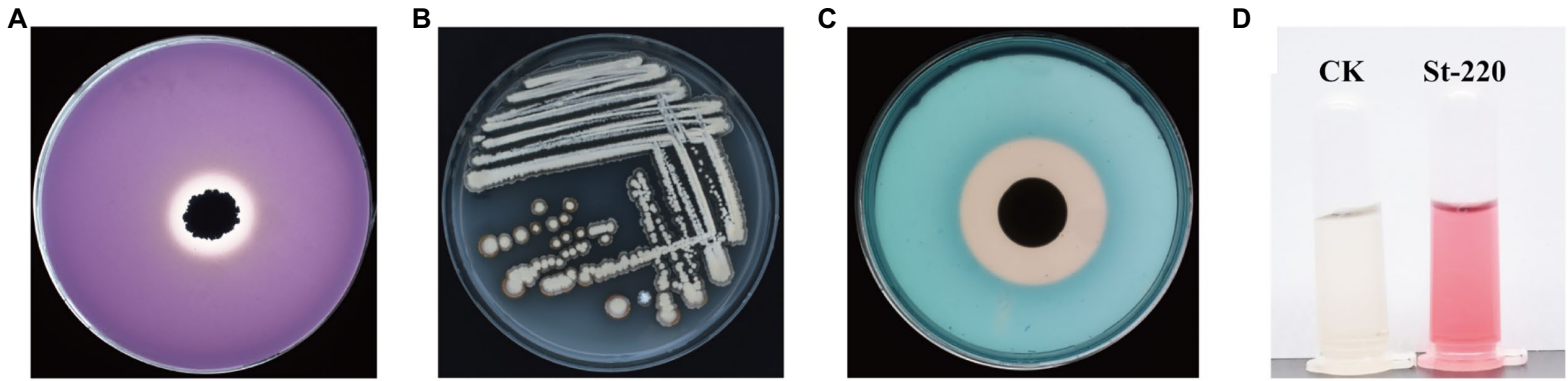
Evaluation of strain St-220 for key traits related to direct plant growth-promotion. **(A)** Qualitative phosphate solubilization assay. **(B)** Biological nitrogen fixation activity assay. **(C)** Siderophores qualitative production assay. **(D)** Production of indole acetic acid activity assay.

To explore the potential mechanism of St-220 on plant growth-promoting activity, four biological characteristics of strain St-220 were tested. In phosphate solubilizing activity assay, a distinct circle around the colony was generated after 7 days of strain St-220 growing on PVK medium (Figure 3A), demonstrating that strain St-220 possessed phosphate solubilizing activity. Strain St-220 was able to grow on Ashby’s nitrogen-free medium after 3 successive transfers suggesting nitrogen-fixing activity (Figure 3B). The siderophore generating carrier activity of strain St-220 was indicated by the creation of a prominent yellow halo surrounding the colony after 7 days of growth in Chrome Azurol Blue agar (Figure 3C). The IAA production activity of strain St-220 was also determined (Figure 3D). Strain St-220 produced maximum 30.40 μg/ml of IAA at 7 DAI, according to a standard curve based on series dilution [y = 0.0094x + 0.0430 (R^2^ = 0.9735, where y is the absorbance value at wavelength of 530 nm, x is the concentration of IAA)] (Supplementary Figure S1).

### Plant growth-promotion activity of St-220 on *Salvia miltiorrhiza*

**Figure 4:**
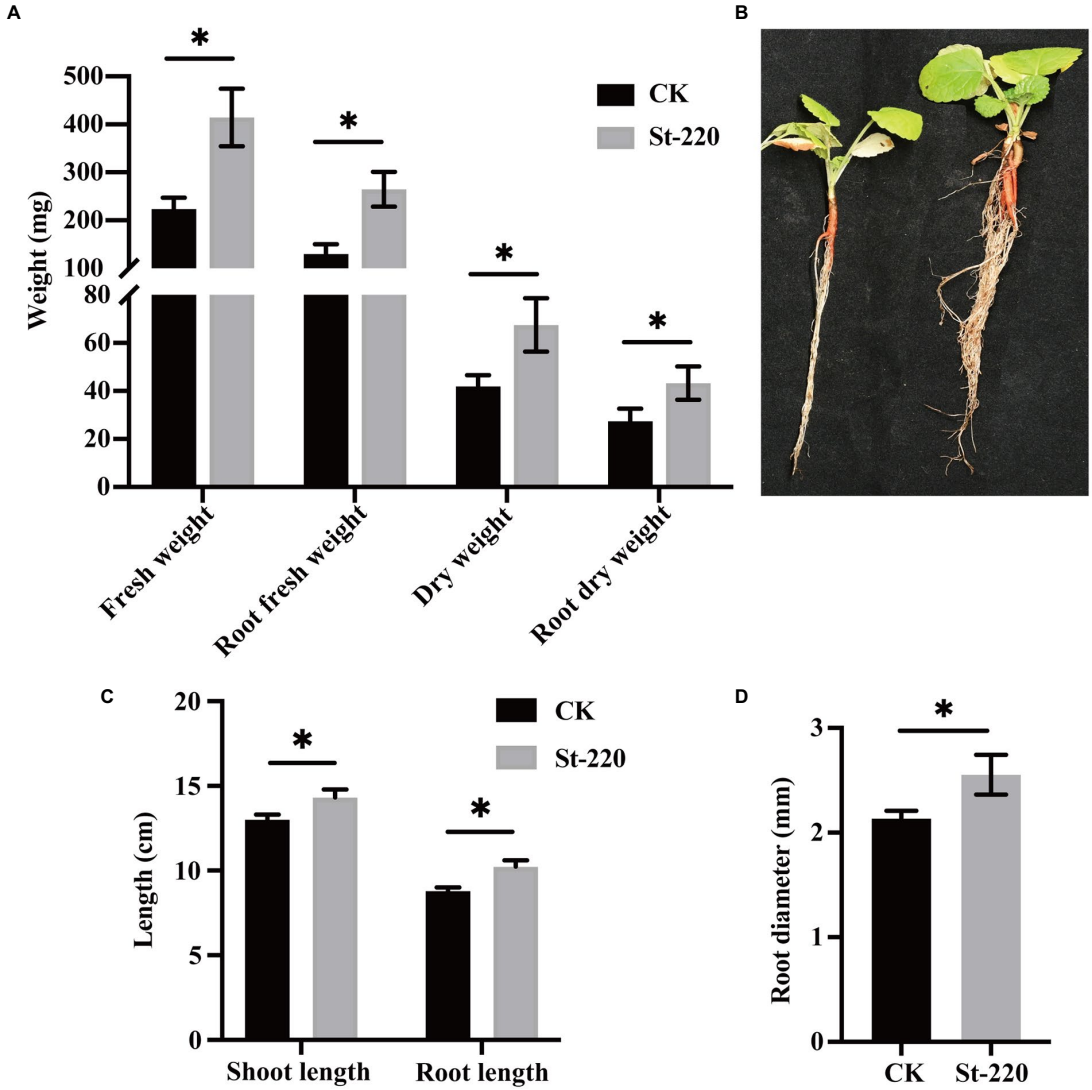
The growth-promoting effect of St-220 on *Salvia miltiorrhiza* seedlings. The growth-promoting activity of strains St-220 was measured under greenhouse conditions, and the data were recorded at 40 days after inoculation. **(A)** the biomass of *Sa. miltiorrhiza* seedlings. **(B)** overall development of *Sa. miltiorrhiza* seedlings inoculated with sterile water (left) and St-220 (right). The shoot height, root length **(C)** and root diameter **(D)** of *Sa. miltiorrhiza* seedlings inoculated with sterile water and cell suspension of St-220. Data are mean ± SE (*n* = 10). Means were considered statistically different when *p* < 0.05, Bars with ∗ above are statistically different, ns above are not statistically different.

To investigate the growth-promoting impact of strain St-220 on *Sa. miltiorrhiza*, a greenhouse experiment was performed and the plant traits was assessed at 40 DAI. The results suggested that strain St-220 was able to stimulate *Sa. miltiorrhiza* growth in contrast to non-inoculated plants, since it exhibited increases in shoot height and fresh weight in roots and plants. St-220 significantly increased the root fresh weight, total fresh weight, total dry weight and root dry weight of *Sa. miltiorrhiza* seedlings by 85.22%, 105.50%, 60.88%, and 36.72%, respectively (Figure 4A). Shoot length and root length also showed an increase (Figures 4B-D).

### Identification of St-220 strain

**Figure 5:**
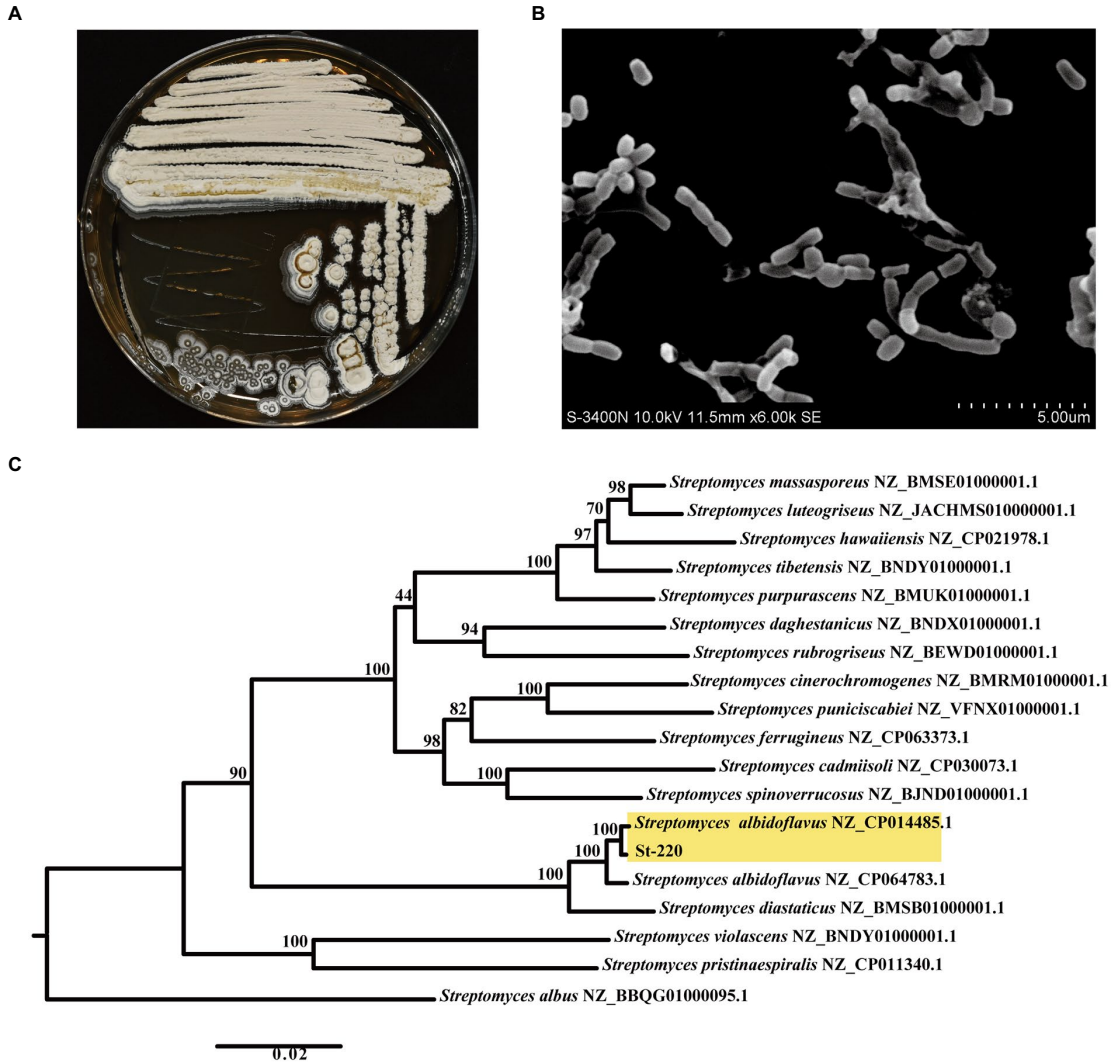
Morphological and molecular identification of strain St-220. **(A)** Colony morphology of the strain St-220 on PDA medium after 14 days of incubation at 28°C. **(B)** Spores of St-220 observed under scanning electron microscope after incubated on PDA medium for 14 days at 28°C. **(C)** A phylogenetic tree using the maximum likelihood method based on the sequences of 16S rDNA and 5 housekeeping genes with 1,000 bootstraps.

After 2-week incubation on PDA, the colony morphology of St-220 revealed a firm surface with white aerial mycelia and faintly whitish-yellow spores (Figure 5A), which is consist with typical morphological characteristics of the *Streptomyces* genus. Both substrate and aerial mycelia were grown well without fragmentation. The flexuous spore chains formed by cylindrical spores were observed under our scanning electron microscope observation (Figure 5B).

The 16S rDNA sequence of St-220 was amplified by PCR and sequenced, and in turn searched in the EzTaxon database, and the strains with high similarity were screened. The sequences of the 16S rDNA and 5 housekeeping genes (*atpD*, *gyrB*, *recA*, *rpoB*, and *trpB*) were concatenated and used to construct a phylogenetic tree using the Maximum-Likelihood method with 1,000 bootstraps. The results suggested that strain St-220 and *St. albidoflavus* clustered into a same clade (Figure 5C). To further confirm our result, the Average Nucleotide Identity (ANI) and DNA–DNA hybridization (DDH) values between St-220 and other 13 *Streptomyces* strains were calculated. The genome of *St. albidoflavus* showed the highest ANI and DDH value of 98.87% and 93.90, among the test strains, respectively, (Supplementary Table S3), which was greater than the threshold value of 95% ~ 96% and 70 for species delineation (Richter and Rosselló-Móra, 2009). Altogether, strain St-220 is recognized as a new member of the *St. albidoflavus* species.

### Genome features of St-220

To have a deep insight in the molecular mechanisms of inhibitory effect and plant growth-promoting, the whole genome of St-220 was sequenced and analyzed. After adapter trimming, the reads were *de novo* assembled into 175 contigs. The genome size of St-220 is 7,310,412 bp with G + C content of 73.41%. The whole genome sequence for St-220 have been deposited in the GenBank database with accession number of JAMFMD000000000. Genomic analysis revealed that the genome of St-220 contained 6,327 CDSs accounting for ~85.43% of the genome (Table 1).

**Table 1:** Genome features of *Streptomyces albidoflavus* St-220.

Features	Genome
Genome size (bp)	7,310,412
Gene Number	6,327
Gene total length	6,245,418
G + C content (%)	73.58
Genome coverage	85.43
Contings	175
Contings N50 (bp)	71,800
Number of ORFs	6,327
tRNA genes	65
rRNA genes	6
CRISPRs	48
Genomic island	10
Genome accession number	JAMFMD000000000

**Figure 6:**
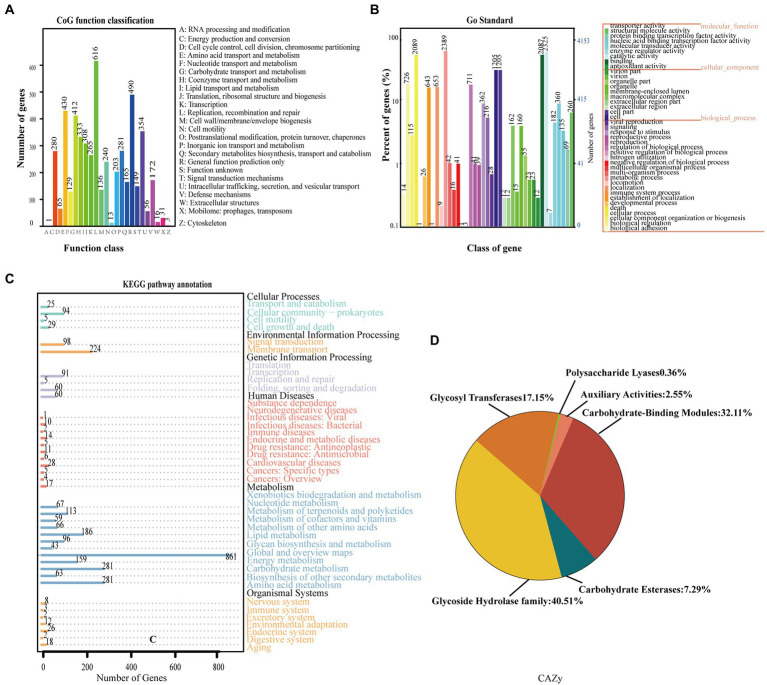
Analysis of genome structure and metabolic pathway of strain *Streptomyces albidoflavus* St-220. **(A)** COG annotation of strain *St. albidoflavus* St-220. genome. **(B)** GO functional categories of *St. albidoflavus* St-220. **(C)** Pathway annotation of strain *St. albidoflavus* St-220 genome according to the KEGG database. The vertical axis represented the level two classification of KEGG pathway. The horizontal axis represented the gene number annotated in this classification. Different colors of the columns represented different classifications of KEGG pathway. **(D)** Gene count distributions of carbohydrate-active enzyme (CAZy) families.

Functional analysis revealed that 5,148, 4,152, 4,798 out of the 6,244 identified CDSs were assigned to COG, GO, and KEGG categories, respectively. In COG categories, the highest ratio the metabolism process was assigned gene numbers with ratio of 36.77%, followed by the category of information storage and processing (17.89%), and the category of cellular processes and signaling (28.61%; Figure 6A). Gene ontology analysis revealed that the category of biological process contained the most GO terms and genes (8,166), followed by molecular function (5,437) and cellular component (2,872; Figure 6B). KEGG pathway analysis showed that the metabolism pathway had the most genes involved, followed by the pathway of environmental information processing (Figure 6C). Additionally, 274 genes were identified in CAZy database and classified into six families. A total of 111 proteins were predicted as belonging to the Glycoside Hydrolase family, of which 88 to Carbohydrate-Binding Modules, 47 to Glycosyl Transferases, 20 to Carbohydrate Esterases, 7 to Auxiliary Activities, and 1 to the Polysaccharide Lyases family (Figure 6D).

### Genome analysis of secondary metabolite clusters

**Figure 7:**
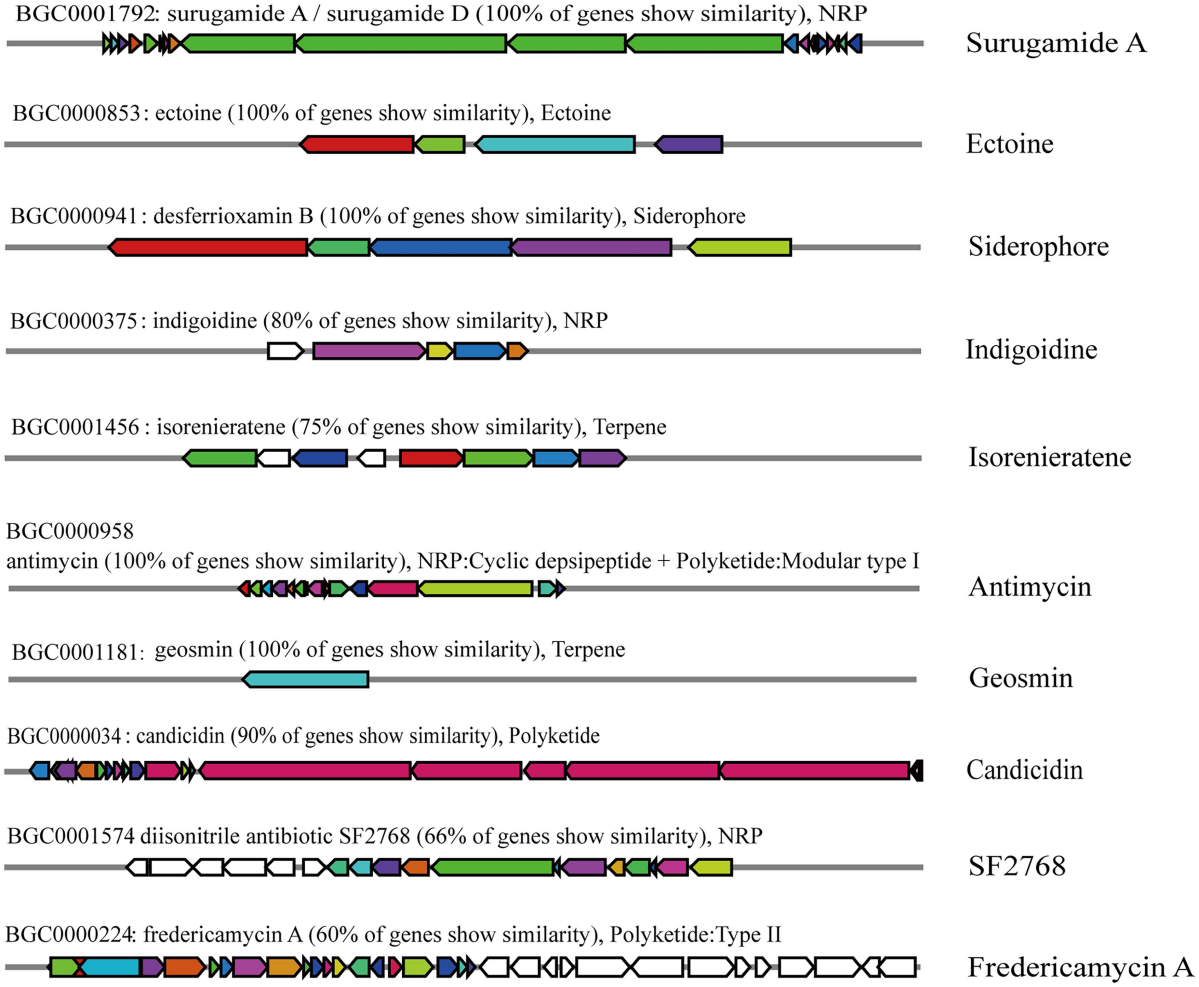
Genome-wide analysis of gene clusters related to the biosynthesis of secondary metabolites using the online antiSMASH v6.0 software.

In our genome mining analysis, the strain St-220 was predicted on produce a plenty of secondary metabolites. By using the antiSMASH, 21 gene clusters for secondary metabolites biosynthesis were predicted and found located in the chromosome of St-220 (Supplementary Table S4), of which 10 gene clusters were involved in the biosynthesis of metabolites antimicrobial activities including ectoine, desferrioxamine B, surugamide A, antimycin, geosmin, indigoidine, isorenieratene, and candicidin (Figure 7). Furthermore, the Region 12.1 was predicted as involved in the desferrioxamine B and E biosynthesis (Supplementary Table S4), which participated the removal of excess iron in the environment.

### Genes associated with fungal cell wall degrading enzymes

The genome of strain St-220 harbors 15 genes encoding enzymes involved in chitin degrading, including six β-N-acetyl hexosaminidase, eight chitinases, and one chitosanase. In addition, St-220 has four chitin-binding proteins belonging to the AA10 family, which enhance the binding abilities of enzymes to insoluble substrates. Four genes in the genome of St-220 were further found to encode endo-1, 3-β-glucanase for degradation of glucan (Supplementary Table S5). Moreover, St-220 contains various genes encoding enzymes that play roles in the degradation of cellulose, protein, and lipids (Supplementary Table S5).

### Genes associated with plant growth-promotion

Our genomic analysis identified several genes related to the plant growth-promoting activities of St-220. These genes participated in 3 trp-dependent biosynthesis pathways of indole-3-acetic acid, including the indole acetamide (IAM), the tryptamine (TAM) and the indole-acetonitrile (IAN) pathways. In the IAM pathway, tryptophan is converted to IAM by tryptophan monooxygenase enzyme, and then amidase enzyme converts IAM to IAA. Nine encoding genes associated with the IAM pathway were found in the St-220 chromosome, of which six encoding tryptophan 2-monooxygenase and three encoding amidas (Supplementary Table S6). In the TAM pathway, tryptophan is firstly converted to TAM, then amine oxidase converts TAM to indole-3-acetaldehyde (IAAld), and finally IAAd is converted to IAA by aldehyde dehydrogenase. Two genes encoding monoamine oxidase and four genes encoding aldehyde dehydrogenase were found to be present in the genome of St-220. The fact that St-220 harbors two separate pathways for IAA biosynthesis suggested that the IAA production plays a role in life maintenance and plant growth-promoting activity. Moreover, St-220 also contains a gene encoding putative1-aminocyclopropane-1-carboxylic acid (ACC) deaminase involved in the decomposition of ACC (Supplementary Table S6) and we made a case that the St-220 could improve the ability of plants to survive under stress conditions by inhibiting ethylene synthesis.

The genome of strain St-220 contains multiple genes involved in the degradation of inorganic polyphosphates and the dissolution of organic phosphates, including a *ppx* gene encoding exopoly phosphatase, a *ppa* gene encoding inorganic pyrophosphatase, and three *phoD* gene*s* encoding alkaline phosphatase. Furthermore, a *pstABCS* cluster involved in the transport and degradation of phosphonates is found in the chromosome of St-220 (Supplementary Table S7).

The genome of Strain St-220 contains one nitrogen fixation protein NifU, and an ammonium transporter protein that was involved in the ability of nitrogen fixation. The strain St-220 genome also contains nine nitrate reductase genes (Supplementary Table S8).

The St-220 genome harbors plenty of genetic elements involved in siderophore biosynthesis and iron complex transport (Supplementary Table S9). Moreover, one cluster involved in siderophore biosynthesis is also present in the chromosome sequence of St-220.

## Discussion

Root rot disease caused by *F. oxysporum* is one of the most severe soil-borne disease worldwide, and also the main constraint of *Sa. miltiorrhiza* production in China. In the present study, an actinomycete strain St-220 with biocontrol activity was isolated from roots of *Sa. miltiorrhiza* and identified as *Streptomyces albidoflavus.* The strain showed inhibition rate of 53.40% against *F. oxysporum* in the dual culture assay and control effect of 77.33% on root rot disease incidence in greenhouse condition. In addition, *St. albidoflavus* St-220 strain also promoted the growth of *Sa. miltiorrhiza* by increasing biomass including total fresh weight, root fresh weight, total dry weight and root dry weight, as well as shoot and root length. These results indicate that *St. albidoflavus* St-220 is a promising biocontrol agent for the control of root rot disease and biofertilizer for *Sa. miltiorrhiza*.

### *Streptomyces albidoflavus* St-220 have both biological control activity and plant growth-promoting activity

Some *Streptomyces* strains could significantly improve the biocontrol of *Fusarium* root rot disease and promote the growth of plant seedlings (El-Tarabily et al., 2009; Goudjal et al., 2016; Tamreihao et al., 2016; Chen et al., 2021). They were generally identified by three properties: IAA production, the abilities to solubilize phosphate and fix nitrogen, and siderophores production (Vurukonda et al., 2018). IAA is a phytohormone that regulates the growth of plant roots by stimulating the development of root (Lwin et al., 2012), and is also an important trait of plant growth-promoting microorganism. Tomato seedlings significantly increased in fresh and dry weight after treated with IAA producing strain *S. fradiae* (Myo et al., 2019). Phosphorus as a macronutrient is dispensable for plants (Ågren and Weih, 2020). Most of the phosphorus, however, present in the form of insoluble in the soil, and cannot be directly utilized by plants (Rawat et al., 2021). The *Streptomyces* strains with growth-promoting activity can dissolve the insoluble phosphate for plant growth. Inoculation of *Streptomyces* sp. strain 7.1 with inorganic phosphate solubilizing activity significantly increased the fresh weight of roots and stems of rice (Suárez-Moreno et al., 2019). Nitrogen is critical to whole life cycle of plants. The atmospheric nitrogen was transformed into ammonia that could be utilized by plants through nitrogen fixation (Dobbelaere et al., 2003). The siderophores secreted by biocontrol agents could suppress the pathogen and protect plants from pathogen infection by iron-competition and restructuring rhizosphere microbiome (Amano et al., 2011; Yu et al., 2013; Gu et al., 2020). For example, the endophytic *Streptomyces* strains SNL1 and SNL2 producing siderophores have antagonistic activities against *F. oxysporum* f. sp. *cubenese* causing *Fusarium* wilt of banana (Cao et al., 2005). *Streptomyces* can further promote plant mineral nutrient supply by synthesizing siderophores. *Streptomyces* sp. GMKU 3100 producing siderophore was able to promote the growth of rice and mung bean, whereas its siderophore-deficient mutant did not differ from the uninoculated control (Rungin et al., 2012).

Previous studies have revealed that *Streptomyces* strains with above properties showed plant growth-promoting activity. *S. violaceusniger* AC12AB was found to have properties of IAA production, siderophores production, nitrogen fixation and phosphates solubilization. It significantly promoted the potato crop up to 26.8% in field trial (Sarwar et al., 2019). Barley plants inoculated with *S. roseocinereus* MS1B15, a strain with IAA-producing, phosphate solubilizing, and nitrogen-fixing activity, significantly increased shoot and spike length (Chouyia et al., 2020). In this study, application of the St-220 resulted in a significant increase in the biomass of *Sa. miltiorrhiza* seedlings. To elucidate the way that the St-220 promotes the growth, the activities of IAA production, phosphorus solubilization, nitrogen fixation and siderophores production was determined and the synthesis pathway was found in further genomic analysis.

### Genomic analysis revealed the potential antifungal and root growth-promoting mechanism of St-220

The strains of *Streptomyces* genus employ their secondary metabolites as weapons to inhibit phytopathogenic fungi (Amin et al., 2021; Hotta, 2021; Mahasneh et al., 2021; Terra et al., 2021). In this study, genome sequencing revealed that the chromosome of the *St. albidoflavus* St-220 contained 21 conserved biosynthesis gene clusters (BGCs), of which 10 showed high similarities in structure with known BGCs encoding terpenes, non-ribosomal peptides, polyketides, siderophores, and ectoines, which had been proven to participate in the regulation of antimicrobial activities of *Streptomyces* strains (van Bergeijk et al., 2020). Among these compounds, the surugamide A, indigoidine Antimycin and Candicidin SF2768 were found to have antifungal activities (Xu et al., 2017; Santos-Beneit et al., 2022), indicating the potential mechanism of the inhibitory effect of St-220 against *F. oxysporum*.

Chitin, the most important component of fungal cell wall, is the preliminary target that biocontrol agents aim at. *Streptomyces* strains produce chitinases to break through the fungal cell wall. For instance, *S. griseus* secret ChiIS, which belongs to glycosyl hydrolase family 19, to inhibit the growth of *Aspergillus nidulans*, *F. culmorum*, and *S. sclerotiorum* (Hoster et al., 2005). Chitinase produced by *Streptomyces* sp. TK-VL_333 showed antifungal activity against *F. oxysporum* (Kavitha and Vijayalakshmi, 2011). The purified and crude chitinase from *S. luridiscabiei* U05 inhibited the growth of *F. oxysporum* and *Alternaria alternata* (Swiontek Brzezinska et al., 2019). In this study, multiple genes (chitinases, β-N-acetyl hexosaminidase, chitosanase) encoding enzymes involved in chitin degradation were found in the genome of *St. albidoflavus* St-220, indicating that the St-220 deployed several weapons targeting the fungal cell wall for its biocontrol effect.

The genome mining has also confirmed the potential mechanism of *St. albidoflavus* St-220 on promoting root growth of *Sa. miltiorrhiza*. In our greenhouse assay, *St. albidoflavus* St-220 promoted the growth of *Sa. miltiorrhiza* seedlings by increasing the plant biomass, especially the length, diameter, fresh and dry weight of the plant roots (Figure 4). To have a deep perspective on the root promoting mechanism, we tested and found that *St. albidoflavus* St-220 has the biological characteristics involved in plant promoting activity including phosphate solubilization, nitrogen fixation, IAA production and siderophore production. The actinobacterial strains, such as *St*. *alfalfae* strain XN-04, *Streptomyces* sp. NEAU-S7GS2, and *St. chartreusis* strain WZS021, have root growth-promoting activities on cotton, soyabean and sugarcane, respectively, and genes related to IAA, siderophores, phosphate solubilization were identified in their genomes (Wang et al., 2018b, Liu et al., 2019, Chen et al., 2021). In various studies, IAA has been shown to increase plant root size and distribution, as well as root hairs, resulting in higher nutrient uptake from the soil (Datta and Basu, 2000; Gumiere et al., 2014; Liao et al., 2017; Ulrich et al., 2021). A number of encoding genes directly involved in the synthesis of indoleacetic acid were found in the genome of St-220, including two genes encoding monoamine oxidase and four genes encoding aldehyde dehydrogenase. Many plant-associated actinomycetes are able to solubilize phosphorus into a form that can be used by plants by secreting phosphatases and phytases (Suárez-Moreno et al., 2019). In our present study, the genomic sequences of strain St-220 were found to encode acid and alkaline phosphatases, as well as phytases, suggesting a potential root stimulation of St-220. Additionally, 12 genes related to nitrogen fixation were also found in the genome of strain St-220. The nitrogen fixation plays a key role in the promoting activity of biocontrol agents on plant root growth and development (Dobbelaere et al., 2003). Our genome mining confirmed that the *St. albidoflavus* St-220 harbors predicted genes involved in pathways regarding IAA and siderophores production, phosphate solubilization and nitrogen fixation, which may play roles in simulating growth and development of plant roots. Therefore, we speculated that *St. albidoflavus* St-220 promotes plant growth in greenhouse condition through employing genes involved in a variety of metabolites synthesis pathways that may related to growth-promoting effects. Our results revealed the antifungal and growth-promoting activities of the *St. albidoflavus* St-220, and suggested the St-220 could be developed as a promising biological fertilizer.

## Conclusion

Strain St-220 has inhibitory activity against *F. oxysporum* causing root rot disease of *Sa. miltiorrhiza*, and also promotes the growth of *Sa. miltiorrhiza* seedlings. The strain was identified as *St. albidoflavus* by its morphological and molecular characteristics. Our genome sequencing identified many pathways involved in synthesis of secondary metabolites with antifungal and growth-promoting activities, indicating the versatility of St-220 for being developed as a BCA against *Fusarium* wilt of *Sa. miltiorrhiza*.

## Data availability statement

The original contributions presented in the study are publicly available. This data can be found at: https://www.ncbi.nlm.nih.gov/, JAMFMD010000000.

## Author contributions

TW, LG, and LH conceived and designed the experiments. YD and TW performed the experiments and analyzed the data. YD, TW, JJ, YW, CL, KS, JS, BY and CK contributed reagents, materials, and analysis tools. All authors contributed to the article and approved the submitted version.

## Funding

This research was funded by National Natural Science Foundation of China (82104341), the Key Project at Central Government Level: The Ability Establishment of Sustainable Use for Valuable Chinese Medicine Resources (2060302), and Scientific and technological innovation project of China Academy of Chinese Medical Sciences (CI2021A03905).

## Conflict of interest

The authors declare that the research was conducted in the absence of any commercial or financial relationships that could be construed as a potential conflict of interest.

## Publisher’s note

All claims expressed in this article are solely those of the authors and do not necessarily represent those of their affiliated organizations, or those of the publisher, the editors and the reviewers. Any product that may be evaluated in this article, or claim that may be made by its manufacturer, is not guaranteed or endorsed by the publisher.
