# Supplementary material for: Biological control and plant growth promotion properties of Streptomyces albidoflavus St-220 isolated from Salvia miltiorrhiza rhizosphere
Source: Front Plant Sci. 2022 Aug 30;13:976813. doi: 10.3389/fpls.2022.976813 (PMC9468599; doi:10.3389/fpls.2022.976813)
Supplement: Supplementary file 1 [file Data_Sheet_1.docx]

**Supplementary Material**

## Supplementary Figures

**Figure S1.** Standard curve of indole acetic acid content at 530nm.

## Supplementary Tables

**Table S1** Inhibition rate of the isolated actinomycetes against *F.oxysporum.*

| Isolate No. | Inhibition rate (%) |
| --- | --- |
| St-101 | 14.20 |
| St-112 | 45.78 |
| St-120 | 3.96 |
| St-180 | 36.77 |
| St-185 | 40.47 |
| St-193 | 23.06 |
| St-220 | 53.40 |
| St-224 | 33.64 |
| St-249 | 4.48 |
| St-267 | 44.76 |
| St-285 | 23.06 |

**Table S2** Disease inhibition effect of potted disease-resistant actinomycetes.

| Group | incidence rate % | Disease index | Biological control efficiency (%) |
| --- | --- | --- | --- |
| Ck | 0 | 0 | - |
| Fo | 86.67 | 68.00 | - |
| Fo+St | 20.00 | 22.66 | 77.33 |

Note: “-” indicates that the biological control efficiency of the strain St-220 was not detected.

**Table S3** The Average Nucleotide Identity (ANI) and DNA–DNA hybridization (DDH) values between the St-220 genomes and 13 complete genomes of *Steptomyces* evaluated using the ANIb Calculator and Genome-to-Genome Distance Calculator (GGDC) web server version 3.0.

| Strain | ANI (%) | DDH |
| --- | --- | --- |
| St-220 | 100 | 100 |
| *Streptomyces albidoflavus* | 98.87 | 93.90 |
| *Streptomyces peucetius* | 76.42 | 18.50 |
| *Streptomyces cyaneogriseus* | 77.56 | 20.50 |
| *Streptomyces reticuli* | 76.84 | 19.40 |
| *Streptomyces rubrolavendulae* | 78.57 | 12.90 |
| *Streptomyces albus* | 75.40 | 17.00 |
| *Streptomyces leeuwenhoekii* | 77.64 | 20.10 |
| *Streptomyces fodineus* | 75.79 | 18.40 |
| *Streptomyces glaucescens* | 77.23 | 19.30 |
| *Streptomyces alfalfae* | 77.03 | 20.40 |
| *Streptomyces formicae* | 76.25 | 19.80 |
| *Streptomyces alboflavus* | 76.47 | 19.00 |
| *Streptomyces collinus* | 76.96 | 19.50 |

**Table S4** Prediction and functional annotation of [secondary](javascript:;) [metabolite](javascript:;)s.

| Region | Type | **Gene position (bp)** | **Most similar known cluster** | **Similarity** |
| --- | --- | --- | --- | --- |
| Region 2.1 | NRPS | 1-38865 | WS9326 | 7% |
| Region 5.1 | NRPS | 90554-134283 | diisonitrile antibiotic SF2768 | 66% |
| Region 5.2 | terpene, NRPS, NRPS-like | 158193-215225 | valinomycin / montanastatin | 13% |
| Region 8.1 | terpene | 63093-84190 | julichrome Q3-3 / julichrome Q3-5 | 25% |
| Region 9.1 | NRPS, RRE-containing | 60894-169541 | surugamide A / surugamide D | 100% |
| Region 10.1 | ectoine | 110408-120806 | ectoine | 100% |
| Region 12.1 | siderophore | 19815-31635 | desferrioxamin B and E | 100% |
| Region 13.1 | NRPS-like | 7126-50956 | indigoidine | 80% |
| Region 13.2 | RiPP-like, terpene | 71084-100086 | isorenieratene | 75% |
| Region 14.1 | siderophore | 90614-105645 | ficellomycin | 5% |
| Region 16.1 | lanthipeptide-class-iii | 54082-76661 | AmfS | 80% |
| Region 17.1 | T1PKS | 1-100494 | tautomycin | 10% |
| Region 17.2 | T3PKS | 105455-136069 | herboxidiene | 12% |
| Region 18.1 | T1PKS, NRPS, | 48112-135907 | antimycin | 100% |
| Region 31.1 | terpene | 54180-76465 | geosmin | 100% |
| Region 32.1 | RRE-containing, NRPS | 43840-88631 | surugamide A / surugamide D | 61% |
| Region 33.1 | T2PKS | 42826-86513 | fredericamycin A | 60% |
| Region 47.1 | T1PKS, NRPS | 8668-56522 | SGR PTMs | 100% |
| Region 48.1 | NRPS | 14706-51510 | desotamide | 27% |
| Region 50.1 | terpene | 1-11941 | hopene | 46% |
| Region 51.1 | NRPS-like, T1PKS | 11764-46142 | candicidin | 90% |

**Table S5.** Genes involved in the degradation of chitin, glucan, cellulose, protein and lipids.

| Classification | Gene ID | Gene name | Family | EC | Predicted function |
| --- | --- | --- | --- | --- | --- |
| Chitin-related | GM000929 | *-* | GH18 | 3.2.1.14 | chitinase |
|  | GM002637 | *-* | GH18 | 3.2.1.14 | chitinase |
|  | GM002638 | *-* | GH18 | 3.2.1.14 | chitinase |
|  | GM005087 | *-* | GH18 | 3.2.1.14 | chitinase |
|  | GM006288 | *-* | GH18 | 3.2.1.14 | chitinase |
|  | GM001041 | *-* | GH18 | 3.2.1.14 | exochitinase |
|  | GM001100 | *-* | GH19 | 3.2.1.14 | chitinase |
|  | GM005033 | *-* | GH19 | 3.2.1.14 | chitinase |
|  | GM001042 | *cpbD* | AA10 | - | chitin-binding protein |
|  | GM001456 | *cpbD* | AA10 | - | chitin-binding protein |
|  | GM003109 | *cpbD* | AA10 | - | chitin-binding protein |
|  | GM003387 | *cpbD* | AA10 | - | chitin-binding protein |
|  | GM000579 | *csn* | GH46 | 3.2.1.132 | chitosanase |
|  | GM000981 | *nagZ* | GH3 | 3.2.1.52 | β-N-acetyl hexosaminidase |
|  | GM003303 |  | GH20 | 3.2.1.52 | hexosaminidase |
|  | GM003500 |  | GH20 | 3.2.1.52 | hexosaminidase |
|  | GM003540 |  | GH20 | 3.2.1.52 | hexosaminidase |
|  | GM001307 | *nagZ* | GH20 | 3.2.1.52 | β-N-acetyl hexosaminidase |
|  | GM002200 | *nagZ* | GH3 | 3.2.1.52 | β-N-acetyl hexosaminidase |
| Glucan-related | GM001718 | *-* | GH16 | 3.2.1.39 | endo-1,3-β-glucanase |
|  | GM002795 | *-* | GH16 | 3.2.1.39 | endo-1,3-β-glucanase |
|  | GM004332 | *-* | GH16 | 3.2.1.39 | endo-1,3-β-glucanase |
|  | GM004762 | *-* | GH64 | 3.2.1.39 | endo-1,3-β-glucanase |
| Cellulose-related | GM002002 | *-* | GH6 | 3.2.1.4 | endo-1,4-β-glucanase |
|  | GM002599 | *-* | GH6 | 3.2.1.4 | endo-1,4-β-glucanase |
|  | GM003392 | *-* | GH6 | 3.2.1.4 | endo-1,4-β-glucanase |
|  | GM002269 | *bglX* | GH1 | 3.2.1.21 | β-glucosidase |
|  | GM000202 | *bglB* | GH1 | 3.2.1.21 | β-glucosidase |
|  | GM000989 | *bglB* | GH1 | 3.2.1.21 | β-glucosidase |
|  | GM002143 | *bglX* | GH1 | 3.2.1.21 | β-glucosidase |
|  | GM002148 | *bglX* | GH1 | 3.2.1.21 | β-glucosidase |
|  | GM004547 |  | GH1 |  | β-glucosidase |
|  | GM004761 |  | GH1 |  | β-glucosidase |
|  | GM006045 |  | GH1 |  | β-glucosidase |
|  | GM001683 |  | GH2 | 3.2.1.165 | β-glucosidase |
|  | GM000652 |  | GH2 | 3.2.1.165 | β-glucosidase |
|  | GM001683 |  | GH1 | 3.2.1.23 | β-glucosidase |
|  | GM001573 |  | GH3 | 3.2.1.21 | β-glucosidase |
|  | GM002898 |  | GH3 | 3.2.1.21 | β-glucosidase |
|  | GM000981 |  | GH3 | 3.2.1.21 | β-glucosidase |
|  | GM000988 |  | GH3 | 3.2.1.21 | β-glucosidase |
|  | GM000989 |  | GH3 | 3.2.1.21 | β-glucosidase |
|  | GM002200 |  | GH3 | 3.2.1.21 | β-glucosidase |
|  | GM004001 |  | GH3 | 3.2.1.21 | β-glucosidase |
| Protein-related | GM000158 |  |  |  | Zn-dependent protease |
|  | GM000314 | *ClpA* |  |  | ATP-dependent Clp protease |
|  | GM000463 |  |  |  | Periplasmic serine protease |
|  | GM000735 | *YfgC* |  |  | Outer membrane metalloprotease |
|  | GM000741 |  |  |  | Serine protease |
|  | GM001103 |  |  |  | protease |
|  | GM001192 |  |  |  | Zn-dependent protease |
|  | GM001258 |  |  |  | Serine protease |
|  | GM001275 | *CtpA* |  |  | C-terminal processing protease |
|  | GM001371 |  |  |  | Zn-dependent protease |
|  | GM001462 |  |  |  | Regulator of protease activity HflC |
|  | GM001655 |  |  |  | Periplasmic serine protease |
|  | GM001743 |  |  |  | ATP-dependent Zn proteases |
|  | GM001796 | *ClpA* |  |  | ATP-dependent Clp protease |
|  | GM001806 | *ClpA* |  |  | ATP-dependent Clp protease |
|  | GM001942 |  |  |  | Serine protease |
|  | GM001973 |  |  |  | Membrane protease activity |
|  | GM001974 |  |  |  | Regulator of protease activity HflC |
|  | GM002086 |  |  |  | Tricorn protease |
|  | GM002231 |  |  |  | Serine protease |
|  | GM002270 |  |  |  | Zn-dependent protease |
|  | GM002484 |  |  |  | Serine protease |
|  | GM002970 |  |  |  | Serine protease |
|  | GM003016 |  |  |  | Cysteine protease |
|  | GM003440 |  |  |  | M6 metalloprotease family |
|  | GM003867 |  |  |  | CAAX protease family |
| Lipids-related | GM000337 |  |  |  | lipase |
|  | GM000666 |  |  |  | Lysophospholipase |
|  | GM000970 |  |  |  | lipase |
|  | GM001306 |  |  |  | Lysophospholipase |
|  | GM001441 |  |  |  | Lysophospholipase |
|  | GM001457 |  |  |  | Lipase EstA |
|  | GM001639 |  |  |  | Lysophospholipase |
|  | GM000643 |  |  |  | Lysophospholipase L1 |
|  | GM001719 |  |  |  | Lysophospholipase |
|  | GM002670 |  |  |  | Lysophospholipase |
|  | GM002794 |  |  |  | Lysophospholipase L1 |
|  | GM002875 |  |  |  | Lysophospholipase |
|  | GM004643 |  |  |  | Lipase |
|  | GM005352 |  |  |  | Lysophospholipase L1 |
|  | GM006060 |  |  |  | Lipase |

**Table S6.** Genes involved in the modulation of plant hormones.

| Gene ID | Gene name | Product | Pathway |
| --- | --- | --- | --- |
| GM004532 |  | 1-aminocyclopropane-1-carboxylate deaminase | ACC catabolism |
| GM000695 | *trpA* | tryptophan synthase alpha chain | L-tryptophan production |
| GM003079 | *trpA* | tryptophan synthase alpha chain |  |
| GM003078 | *trpB* | tryptophan synthase beta chain |  |
| GM003077 | *trpC* | indole-3-glycerol phosphate synthase |  |
| GM003073 | *trpE* | anthranilate synthase component I |  |
| GM005864 | *trpD* | anthranilate phosphoribosyltransferase |  |
| GM000909 |  | tryptophan 2-monooxygenase | IAA production; IAM pathway |
| GM000335 |  | tryptophan 2,3-dioxygenase |  |
| GM000695 |  | tryptophan synthase alpha chain |  |
| GM002941 |  | tryptophan 2,3-dioxygenase |  |
| GM002942 |  | tryptophan 2,3-dioxygenase |  |
| GM003752 | *amiE* | amidase |  |
| GM001992 |  | penicillin amidase |  |
| GM002552 | *pncC* | nicotinamide-nucleotide amidase |  |
| GM003850 | *aofH* | monoamine oxidase | IAA production; TAM pathway |
| GM003851 | *aofH* | monoamine oxidase |  |
| GM003945 |  | aspartate-semialdehyde dehydrogenase |  |
| GM003478 | ALDH | aldehyde dehydrogenase (NAD+) |  |
| GM004364 | sauS | sulfoacetaldehyde dehydrogenase |  |
| GM005101 | betB, | betaine-aldehyde dehydrogenase |  |

**Table S7.** Genes involved in phosphate solubilization and transport.

| Gene ID | Gene name | Product | Pathway |
| --- | --- | --- | --- |
| GM004322 | *ppx-gppA* | exopolyphosphatase | Degradation of inorganic  polyphosphates |
| GM005308 | *ppa* | inorganic pyrophosphatase |  |
| GM003048 | *phoD* | alkaline phosphatase | Organic phosphate  solubilization |
| GM003740 | *phoD* | alkaline phosphatase D |  |
| GM003795 | *phoD* | alkaline phosphatase D |  |
| GM004522 | *phoH* | phosphate starvation-inducible protein PhoH |  |
| GM002050 |  | phosphinothricin acetyltransferase |  |
| GM004809 | *phoU* | phosphate transport system protein | Phosphate transport |
| GM000383 | *pstS* | phosphate transport system substrate-binding protein |  |
| GM000382 | *pstC* | phosphate transport system permease protein |  |
| GM000381 | *pstA* | phosphate transport system permease protein |  |
| GM000380 | *pstB* | phosphate transport system ATP-binding protein |  |

**Table S8.** Genes involved in Nitrogen fixation.

| Gene ID | Gene name | Product | Pathway |
| --- | --- | --- | --- |
| GM001887 | *iscU* | nitrogen fixation protein NifU and related proteins | Nitrogen fixation |
| GM001888 | *iscU* | nitrogen fixation protein NifU |  |
| GM005619 | *iscU* | ammonium transporter |  |
| GM005618 | *glnB* | nitrogen regulatory protein P-II 1 | Nitrate  reductase |
| GM004089 | *narV* | nitrate reductase gamma subunit |  |
| GM004090 | *entB* | nitrate reductase molybdenum cofactor assembly chaperone |  |
| GM004091 | *narH* | nitrate reductase |  |
| GM004092 | *narG* | nitrate reductase |  |
| GM004928 | *narH* | nitrate reductase |  |
| GM004929 | *narH* | nitrate reductase |  |
| GM004930 | *entB* | nitrate reductase molybdenum cofactor assembly chaperone |  |
| GM004931 | *narV* | nitrate reductase gamma subunit |  |

**Table S9.** Genes involved in iron transport and siderophore production.

| Gene ID | Gene name | Product | Pathway |
| --- | --- | --- | --- |
| GM007018 |  | ferric iron reductase | Ferric iron reduction |
| GM000291 | *entF* | enterobactin synthetase component F |  |
| GM005665 | *entA* | 2,3-dihydro-2,3-dihydroxybenzoate dehydrogenase | Siderphore production |
| GM005664 | *entE* | 2,3-dihydroxybenzoate-AMP ligase |  |
| GM001325 | *entB* | bifunctional isochorismate lyase / aryl carrier protein |  |
| GM000286 |  | iron complex transport system ATP-binding protein |  |
| GM005665 |  | iron complex transport system permease protein |  |
| GM000645 |  | iron complex transport system substrate-binding protein |  |
| GM005667 |  | iron complex transport system permease protein |  |
| GM000720 |  | iron complex transport system substrate-binding protein |  |
| GM000649 |  | iron complex transport system ATP-binding protein |  |
| GM000717 |  | iron complex transport system ATP-binding protein |  |
| GM000284 |  | iron complex transport system permease protein |  |
| GM000285 |  | iron complex transport system permease protein |  |
| GM001064 |  | iron complex transport system substrate-binding protein |  |
| GM001062 |  | iron complex transport system ATP-binding protein |  |
| GM000647 |  | iron complex transport system permease protein |  |
| GM000648 |  | iron complex transport system permease protein |  |
| GM001135 |  | iron complex transport system ATP-binding protein |  |
| GM001203 |  | iron complex transport system ATP-binding protein |  |
| GM000718 |  | iron complex transport system permease protein |  |
| GM001134 |  | iron complex transport system substrate-binding protein |  |
| GM001201 |  | iron complex transport system substrate-binding protein |  |
| GM001972 |  | iron complex transport system ATP-binding protein |  |
| GM000719 |  | iron complex transport system permease protein |  |
| GM001063 |  | iron complex transport system permease protein |  |
| GM002576 |  | iron complex transport system ATP-binding protein |  |
| GM002760 |  | iron complex transport system substrate-binding protein |  |
| GM004852 |  | iron complex transport system substrate-binding protein |  |
| GM002759 |  | iron complex transport system ATP-binding protein |  |
| GM001133 |  | iron complex transport system permease protein |  |
| GM003760 |  | iron complex transport system substrate-binding protein |  |
| GM004966 |  | iron complex transport system substrate-binding protein |  |
| GM001202 |  | iron complex transport system permease protein |  |
| GM003763 |  | iron complex transport system ATP-binding protein |  |
| GM002757 |  | iron complex transport system permease protein |  |
| GM003761 |  | iron complex transport system permease protein |  |
| GM004850 |  | iron complex transport system ATP-binding protein |  |
| GM005664 |  | iron complex transport system ATP-binding protein |  |
| GM005665 |  | iron complex transport system permease protein |  |
| GM005666 |  | iron complex transport system permease protein |  |
| GM005667 |  | iron complex transport system substrate-binding protein |  |
